# Rapidly Progressing Encephalopathy in a 49-Year-Old Female: Creutzfeldt-Jakob Disease

**DOI:** 10.7759/cureus.101665

**Published:** 2026-01-16

**Authors:** Anupama Ancha, Jyothi R Patri

**Affiliations:** 1 Department of Gastroenterology, Baylor Scott & White Medical Center, Temple, USA; 2 Department of Family Medicine, Heritage Valley Family Medicine Residency Program, Beaver Falls, USA; 3 Department of Family Medicine, Philadelphia College of Osteopathic Medicine (PCOM) and Duquesne School of Medicine, Pittsburgh, USA

**Keywords:** cjd, creutzfeldt jacob disease, encephalopathy, prion, tau protein

## Abstract

Creutzfeldt-Jakob Disease (CJD) is a rare neurological disorder. We present the case of a 49-year-old woman brought to the hospital with a rapid decline in daily activities. Her symptoms progressed rapidly over two months. She was evaluated in an outside facility and was diagnosed with conversion disorder. All the initial workup results for the patient were unremarkable. After she spiked a fever, we analyzed her cerebrospinal fluid for prions. The test was positive for tau and 14-3-3 proteins. For a second opinion, the University of San Francisco Prion Research reviewed the case, confirmed CJD, and reported a negative paraneoplastic antibody test. The patient's condition deteriorated with worsening spasticity and aphasia. She had a percutaneous endoscopic gastrostomy tube placed for nutrition and was transferred to life under hospice care with a do-not-resuscitate status per her family's request. This case highlights that CJD should be considered in differential diagnoses in patients with progressive encephalopathy, impaired coordination, visual changes, and mental decline to exclude a curable entity.

## Introduction

Creutzfeldt-Jakob Disease (CJD) is a rare and fatal neurological disorder with an incidence of one case per million per year, with death occurring in 70% of the patients within the first year. There are four main etiologies to consider when classifying the disease: sporadic, familial, variant, and iatrogenic [1]. Familial etiology is CJD in the patient plus definite or probable CJD in a first-degree relative and/or neuropsychiatric disorder plus disease-specific prion protein (PrP) gene mutation [2]. Iatrogenic etiology is a progressive cerebellar syndrome in a recipient of human cadaveric-derived pituitary hormone or sporadic CJD with a recognized exposure risk (e.g., antecedent neurosurgery with dura mater implantation) [2]. Sporadic CJD is the result of a transformation of the cellular prion protein (PrPc) gene into the PrP scrapie isoform by an unknown process, ultimately leading to disease [1]. The initial presentation of CJD may include rapidly progressive encephalopathy, impaired coordination, and/or visual changes, among various symptoms. As the disease progresses, neurological deterioration becomes more pronounced, as involuntary movements, blindness, and coma can present within a few months. A definitive diagnosis is made with a brain biopsy or, more often, with a postmortem autopsy. Therefore, diagnosing CJD can be extremely difficult and relies heavily on clinical observation and the exclusion of other diseases. This case report highlights a unique presentation of CJD and emphasizes the importance of including CJD on the list of differentials for a wide variety of neurologic and psychiatric concerns.

Although the disease is incurable, it is a diagnosis that should be considered and excluded in hopes of identifying another curable cause. In this case report, we discuss a 49-year-old woman recently diagnosed with conversion disorder at the time of presentation at an outside facility who presented with a progressive decline in neurological function over two months.

## Case presentation

A 49-year-old white woman presented with a history of rapidly declining function over two months. The patient explained that she had also been experiencing an inability to eat and bowel and bladder incontinence for the past four days. She added that she was recently diagnosed with conversion disorder at an outside facility after laboratory investigations, magnetic resonance imaging (MRI), and cerebrospinal fluid (CSF) analysis results were unremarkable. She had an unremarkable past medical and surgical history without any risk factors for CJD apart from an ear infection two months ago. She has no known allergies to medications. Her family history was adverse for similar presentations.

Physical examination revealed stable vital signs. On general examination, we noted agitation. The neurological examination showed a conscious female patient who did not track well, withdrew to pain, was aphasic, did not follow commands, had 1+ reflexes, had no clonus, had truncal tremors present, and had spastic movements of the lower extremities. After a thorough evaluation, the patient was admitted for an altered mental status of unknown etiology.

Investigations, results, hospital course

The diagnostic workup included a computed tomography (CT) scan, MRI, electroencephalogram (EEG), and routine laboratory studies. The CT scan was negative for intracranial abnormality, the MRI was unremarkable, and the EEG was normal. 

After admission, we ruled out a possible tumor by obtaining a chest X-ray and abdomino-pelvic CT scan, which revealed nothing remarkable. The patient was treated with intravenous steroids for a potential flare-up of an immunological disease without resolution of symptoms. Psychiatry was consulted with the working diagnosis of conversion disorder in mind, and lorazepam was prescribed as needed. On the third day of hospitalization, the patient developed a fever (101.2℉), prompting consultation with an infectious disease specialist. We started vancomycin for prophylaxis. Repeated sepsis workups were conducted, considering the many possible etiologies, including blood, CSF, and urine cultures. Although the cultures returned negative, the patient continued to have intermittently spiking fevers throughout the next few days. Simultaneously, the patient was neurologically deteriorating rapidly and exhibited increased myoclonus, a poor prognostic sign. At this time, her CSF was analyzed for potential signs of CJD. The enzyme-linked immunoassay test for Tau protein was positive at 12,218 pg/mL (reference limit is <1200 pg/mL). Her CSF was also positive for the 14-3-3 protein. After careful review of these CSF results, the diagnosis of CJD was made.

The case was reviewed, and subsequently, the diagnosis was confirmed by the University of San Francisco Prion Research Center. The Center also reported a negative paraneoplastic antibody test. The patient and the family decided to withdraw all care and change her code status to "do not resuscitate." Supportive measures were taken to ensure her comfort. A percutaneous endoscopic gastrostomy tube was placed for nutrition, and all medications were discontinued except for clonidine and acetaminophen as needed. The patient was transferred to a subacute end-of-life care center for hospice care.

## Discussion

**Figure 1:**
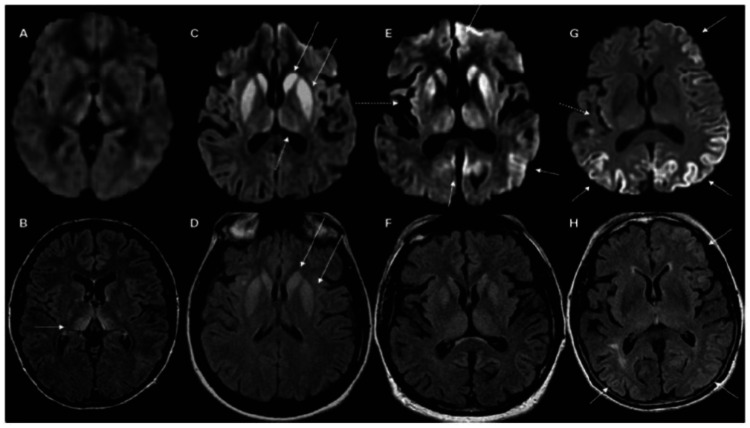
Abnormal MRI findings in sCJD; C, D: Subcortical lesions; E, F: Both cortical and subcortical lesions; G, H: Cortical lesions. A, C, E, and G are DWI images that are more prominent, while B, D, F, and H are FLAIR images. Original figure attributed to Staffaroni AM, Elahi FM, McDermott D, et al.: Neuroimaging in dementia. Semin Neurol. 2017, 37:510-537. 10.1055/s-0037-1608808 and permission was obtained from the original publisher. MRI: magnetic resonance imaging; sCJD: Sporadic Creutzfeldt Jakob Disease; DWI: diffusion-weighted imaging; FLAIR: fluid-attenuated inversion recovery.

CJD can be suspected in many cases through the help of brain imaging, such as MRI, EEG, and CSF analysis. A diffusion-weighted brain MRI may be one of the most helpful diagnostic tests [3]. Restriction of diffusion in the cortex, basal ganglia, and thalamus indicates sporadic CJD (Table 1, Figure 1) [4,5]. Parenchymal swelling is typically absent and may lead to other diagnoses such as encephalitis [5]. Although limited in sensitivity, the EEG may show periodic sharp wave complexes [6]. Even as the role of EEG in diagnosing CJD has declined, it is still helpful in ruling out other disorders, an essential aspect of the workup in CJD. The gold standard for the diagnosis of CJD is a brain biopsy. However, studies have shown that many antemortem biopsies are inconclusive or non-diagnostic. Postmortem biopsies may have a higher diagnosis rate due to better visualization and access to diseased portions of the brain. A recent emerging test that can potentially change the course of CJD diagnosis is the real-time quaking-induced conversion (RT-QuIC). The test can detect the presence of prion proteins without the need for a brain tissue specimen. Usually, a CSF specimen is used, but other specimens may be used in the future. Although many factors may alter RT-QuIC results, there is great potential for improvement, leading to a more accessible recognition mode of CJD. For this reason, a higher burden is placed on clinical suspicion and diagnosis [2].

**Table 1:** Differential diagnosis for CJD CJD: Creutzfeldt-Jakob disease; PCR: polymerase chain reaction; FLAIR: fluid-attenuated inversion recovery; MRI: magnetic resonance imaging.

Condition	Signs/symptoms	Diagnosis	CJD vs. Differential Diagnosis
Hypoxic-ischemic and hypoglycemic encephalopathy and hypoglycemic encephalopathy	A patient's history is essential as signs of cardiorespiratory collapse or low blood glucose level can differentiate them	Increased signal intensity in regions of the brain that are more metabolically active	
Posterior reversible encephalopathy	Occurs due to vasoconstriction or abnormal autoregulation and leads to a decrease in perfusion to the posterior regions of the brain	Vasogenic edema in the parietal and occipital regions is the classic radiological finding	Visual disturbance and nonspecific symptoms imaging pattern and reversibility of the disease will distinguish it from CJD
Herpes encephalitis	Common cause of viral encephalitis is fatal, and presents rapidly progressing nonspecific symptoms that only affect limbic structures, most commonly the temporal lobes	Viral DNA PCR will identify the disease	The basal ganglia are spared, unlike in CJD
Autoimmune encephalitis	It manifests as intractable epilepsy	MRI shows T2 and FLAIR abnormalities in the limbic symptom, and may include the cortex and cerebellum	
Wernicke’s encephalopathy	It is a neurological disorder caused by Vitamin B1 deficiency, classically presents with ophthalmoplegia, ataxia, and altered mental status	MRI will show bilateral and symmetric alteration in signal intensity on MRI typically involving the thalami, periventricular regions of the third ventricle, mamillary bodies, tectal plate, and periaqueductal area	The involvement of the thalamus may imitate the double hockey stick sign, but the sparing of the cortex, nutritional history, and laboratory tests can help diagnose Wernicke's
Wilson's disease	An inherited disorder of impaired copper metabolism that leads to the accumulation of copper in the brain, liver, and eyes	Hyperintensity in the basal ganglia, thalamus, midbrain, and pons on T2/FLAIR MRI images is typically present	Family history, age of presentation, laboratory results, and the lack of involvement of the cortex can point to Wilson's disease as opposed to CJD

Given the importance of ruling out a treatable underlying cause, some differential diagnoses to consider are those with similar clinical symptoms or similar radiological findings (Table 2). In addition, electrolyte abnormalities, Hashimoto thyroiditis, meningitis, infectious encephalitis, B12 deficiency, heavy metal toxicity, and causes of dementia should be considered [1].

**Table 2:** CDC diagnostic criteria for sporadic CJD CDC: Centers for Disease Control and Prevention; CJD: Creutzfeldt-Jakob disease; sCJD: sporadic Creutzfeldt-Jakob disease; PrPsc: prion protein scrapie isoform; EEG: electroencephalogram; CSF: cerebrospinal fluid; DW: diffusion-weighted; FLAIR: fluid-attenuated inversion recovery; MRI: magnetic resonance imaging.

	Definite	Probable	Possible
Signs/symptoms		Presence of rapidly progressive dementia with at least two of the following: myoclonus, visual impairment, cerebellar signs, pyramidal/extrapyramidal signs, and akinetic mutism [2]	Similar symptoms as probable sCJD but of duration less than two years [2]
Diagnosis	Requires tissue confirmation using immunohistochemistry, Western blot, or the presence of PrPsc fibrils, diagnosis is commonly made postmortem [2]	At least one diagnostic finding using laboratory testing and imaging must be present. This includes periodic sharp wave complexes on EEG, 14-3-3 protein on CSF, and hyperintensity in the thalamus (caudate nucleus/putamen) or two cortical regions on either DW or FLAIR images of MRI AND without routine investigations indicating an alternative diagnosis [2]	Negative or atypical results from the three tests AND without routine investigations indicate an alternative diagnosis [2]

Because there is no cure for CJD and death is inevitable within a few months to a year, treatment focuses on supportive and palliative therapy and empiric treatment for other treatable causes. Antipsychotics may be used for agitation, and antiepileptics may be used to treat myoclonus. Empiric therapy may include acyclovir for herpes encephalitis and corticosteroids if an autoimmune condition is suspected [1]. The prognosis is guarded, and the family should be counseled about the prognosis and available supportive treatment modalities.

## Conclusions

CJD is a rare and fatal neurological disease with a rapidly progressive course. The presentation usually involves neurological deficits with or without psychiatric problems. Death can occur in a few weeks to one year. As this case highlights, it is important to maintain a high suspicion for CJD in patients with rapidly progressive neurological decline once more common causes have been excluded. Despite its typical presentation, the diagnostic process and clinical course offer valuable learning points for timely recognition and management.
